# Signaling Toward Reactive Oxygen Species-Scavenging Enzymes in Plants

**DOI:** 10.3389/fpls.2020.618835

**Published:** 2021-02-01

**Authors:** Petr Dvořák, Yuliya Krasylenko, Adam Zeiner, Jozef Šamaj, Tomáš Takáč

**Affiliations:** Department of Cell Biology, Centre of the Region Haná for Biotechnological and Agricultural Research, Faculty of Science, Palacký University, Olomouc, Czechia

**Keywords:** signaling, stress, oxidative stress, plants, mitogen-activated protein kinases, calcium, antioxidant enzymes, reactive oxygen species

## Abstract

Reactive oxygen species (ROS) are signaling molecules essential for plant responses to abiotic and biotic stimuli as well as for multiple developmental processes. They are produced as byproducts of aerobic metabolism and are affected by adverse environmental conditions. The ROS content is controlled on the side of their production but also by scavenging machinery. Antioxidant enzymes represent a major ROS-scavenging force and are crucial for stress tolerance in plants. Enzymatic antioxidant defense occurs as a series of redox reactions for ROS elimination. Therefore, the deregulation of the antioxidant machinery may lead to the overaccumulation of ROS in plants, with negative consequences both in terms of plant development and resistance to environmental challenges. The transcriptional activation of antioxidant enzymes accompanies the long-term exposure of plants to unfavorable environmental conditions. Fast ROS production requires the immediate mobilization of the antioxidant defense system, which may occur *via* retrograde signaling, redox-based modifications, and the phosphorylation of ROS detoxifying enzymes. This review aimed to summarize the current knowledge on signaling processes regulating the enzymatic antioxidant capacity of plants.

## Introduction

Reactive oxygen species (ROS), as unavoidable byproducts of metabolism, have important signaling roles in living organisms under optimal and adverse environmental conditions (Apel and Hirt, 2004; Baxter et al., 2014; Waszczak et al., 2018). ROS are produced from atmospheric oxygen by its partial monovalent reduction, which occurs in the presence of electron donors. Plant ROS are generated mainly by electron transport chains in chloroplasts (Pospíšil, 2016; Foyer, 2018) and mitochondria (Gleason et al., 2011) as well as during photorespiration in peroxisomes (del Río et al., 2006; del Río and López-Huertas, 2016). Apoplastic ROS are produced by plasma membrane-localized NADPH oxidase [NOX, in plants encoded by *RESPIRATORY BURST OXIDASE HOMOLOG (RBOH)* genes; Sagi and Fluhr, 2006], oxalate oxidase (Voothuluru and Sharp, 2013), or by the degradation of spermidine by polyamine oxidase (Geilfus et al., 2015). Apoplastic peroxidases also possess ROS generating capacity (Bindschedler et al., 2006). Only four ROS, namely singlet oxygen (_1_O^2^), superoxide (O_2_^·−^), hydrogen peroxide (H_2_O_2_), and hydroxyl radical (OH^·^), are more abundant and stable. They quickly interconvert, thus providing a high level of functional variability. However, they differ in their stability, reactivity, and ability to be transported across membranes. H_2_O_2_ is the most stable ROS transported actively across membranes by aquaporins (Miller et al., 2010; Mittler, 2017; Smirnoff and Arnaud, 2019).

Depending on the generated ROS concentration, severity of stress, antioxidant capacity, and cellular energetic status, different cellular and physiological outcomes may be obtained. In small concentrations, ROS exert signaling functions, leading to the expression of stress-responsive genes. Upon extensive accumulation, ROS reactivity may cause damaging oxidative effects on lipids, nucleic acids, and proteins, eventually resulting in cell death. Moreover, ROS are involved in strictly regulated programmed cell death (Lehmann et al., 2015; Petrov et al., 2015). A very important feature of ROS signaling is that it can be propagated from cell to cell and transduce signals for long distances in a process known as the ROS wave (Mittler et al., 2011). This is mediated by the interplay between NOX, calcium (Ca^2+^) channels, and oxidative stress-induced Ca^2+^ fluxes (Gilroy et al., 2016). The ROS wave, together with other hormone- or electric signal-mediated signaling mechanisms, is implicated in systemic acquired acclimation, in which the locally generated stress signal is transported into sites not being directly affected by the stress. Systemic acquired acclimation is accompanied by rapid and dramatic transcriptional reprogramming (Zandalinas et al., 2019, 2020).

In addition to plant stress responses, ROS are widely involved in developmental processes (Considine and Foyer, 2014; Xia et al., 2015; Mhamdi and Van Breusegem, 2018), regulating morphogenetic processes in the close interplay of phytohormones such as auxins and cytokinins (Xia et al., 2015; Zwack et al., 2016). Interestingly, auxin and abscisic acid (ABA) can promote ROS production by activating NOX (Joo et al., 2001; Schopfer et al., 2002; Pasternak et al., 2007). In turn, ROS may affect auxin levels (Takáč et al., 2016a) and homeostasis leading to altered shoot branching and leaf rosette shapes (Tognetti et al., 2010).

This review aimed to summarize recent findings on the regulation of major ROS-decomposing enzymes. First, we introduce the major antioxidant enzymes and provide an overview of the mechanisms by which ROS affect signaling in plants. Next, we summarize recent knowledge on the redox regulation of major antioxidant enzymes and elaborate on their modulation by mitogen-activated protein kinase (MAPK) and Ca^2+^ signaling pathways. The transcriptional activation of antioxidant enzymes, including metabolite-driven retrograde signaling, is discussed as well. Finally, we point out the importance of their post-translational regulation by reversible phosphorylation and reactive nitrogen species (RNS).

## Characterization of Key Antioxidant Enzymes

Generally, enzymatic antioxidant capacity inevitably contributes to plant survival in adverse conditions, especially when the stress pressure exceeds the mechanisms preventing ROS overaccumulation. The significance of antioxidant enzymes has been documented many times by genetic studies reporting on the positive correlation between the expression of these enzymes and plant stress tolerance. Contrarily, the deregulation of these enzymes is connected with plant hypersensitivity to stress and programmed cell death (De Pinto et al., 2012).

ROS scavenging is performed enzymatic or *via* non-enzymatic antioxidant defense pathways, which control the regulation of ROS levels through strict compartmentalization (Mignolet-Spruyt et al., 2016; Noctor et al., 2017; Foyer and Noctor, 2020). Non-enzymatic antioxidant defense is mainly mediated by low molecular-weight metabolites such as ascorbate, glutathione, α-tocopherol, carotenoids, and flavonoids (Locato et al., 2017; Smirnoff, 2018; Zechmann, 2018; Muñoz and Munné-Bosch, 2019; Foyer and Noctor, 2020). Superoxide dismutases (SODs), catalases (CATs), ascorbate peroxidases (APXs), dehydroascorbate reductases (DHARs), monodehydroascorbate reductases (MDHARs), and glutathione reductases (GRs) are among the main antioxidant enzyme classes. Furthermore, glutathione peroxidases, peroxidases, and thio-, gluta-, and peroxiredoxins are potent ROS scavengers as well (Dietz, 2011; Kang et al., 2019; Foyer and Noctor, 2020). Within this section, we briefly characterize the key enzymatic antioxidants in plants.

Major enzymatic antioxidants are plastidic, cytosolic, mitochondrial, and peroxisomal SODs, which decompose O_2_^.−^ to H_2_O_2_ (Kliebenstein et al., 1998; Alscher et al., 2002; Pilon et al., 2011). Based on the presence of metal cofactors in their active site, four different SODs are recognized in living organisms, namely FeSOD, MnSOD, NiSOD (not present in higher plants), and Cu/ZnSOD. In the *Arabidopsis thaliana* genome, three *FeSOD* (*FSD1, FSD2*, and *FSD3*), one *MnSOD* (*MSD1*), and three *Cu/ZnSOD* (*CSD1, CSD2*, and *CSD3*; Kliebenstein et al., 1998; Pilon et al., 2011) genes have been identified. Individual SOD isozymes are compartmentalized into mitochondria (MSD1; Morgan et al., 2008), peroxisomes (CSD3; Kliebenstein et al., 1998), cytosol (CSD1 and FSD1; Kliebenstein et al., 1998, Dvořák et al., 2020), the chloroplast stroma (FSD1; Kuo et al., 2013; Dvořák et al., 2020), and thylakoids (CSD2, FSD2, and FSD3; Kliebenstein et al., 1998; Myouga et al., 2008). Recently, it was discovered that FSD1 is also localized to the nucleus (Dvořák et al., 2020). In addition to their antioxidative role during salt, oxidative (Myouga et al., 2008; Shafi et al., 2015; Dvořák et al., 2020), and photooxidative stresses (Myouga et al., 2008; Xing et al., 2013; Gallie and Chen, 2019), SODs also have developmental functions during lateral root growth (Morgan et al., 2008; Dvořák et al., 2020), germination (Dvořák et al., 2020), chloroplast development, and flowering (Rizhsky et al., 2003; Myouga et al., 2008).

CATs are responsible for the detoxification of the overproduced H_2_O_2_, which occurs owing to their kinetic properties (Tuzet et al., 2019). As iron-containing homotetrameric proteins, CATs catalyze the decomposition of H_2_O_2_ to H_2_O and O_2_, predominantly produced during photorespiration. Three genes (*CAT1, CAT2*, and *CAT3*) encoding CATs have been found in the *Arabidopsis* genome. CAT isozymes are localized in peroxisomes (Frugoli et al., 1996; Du et al., 2008) and play important roles under unfavorable conditions for plants. For example, all three CAT isoforms are required for the plant response to photooxidative stress (Vandenabeele et al., 2004; Bueso et al., 2007; Zhang S. et al., 2020). CAT2 is involved in plant responses to heat, heavy metal (Corpas and Barroso, 2017; Ono et al., 2020), cold, and salt stresses (Bueso et al., 2007). CAT3 participates in the drought stress response (Zou et al., 2015), whereas CAT1 is implicated in the drought and salt stress responses (Xing et al., 2007). Surprisingly, H_2_O_2_-induced CAT2, likely together with CAT1 and CAT3, generates a signal promoting autophagy-dependent cell death during plant immune responses (Hackenberg et al., 2013; Teh and Hofius, 2014). In addition, tobacco NbCAT1 is relocalized to nuclei after interaction with CRINKLING- AND NECROSIS-INDUCING PROTEIN 63 (CRN63) secreted by *Phytophthora sojae* under attack as found in the transient assay. This mechanism was reported to regulate pathogen-induced cell death in tobacco (Zhang M. et al., 2015). CATs are involved in root growth (Yang et al., 2019), leaf development and senescence (Mhamdi et al., 2010; Zhang Y. et al., 2020), as well as shoot, ovule, pollen, and seed development (Sharma and Ahmad, 2014; Su et al., 2018; Palma et al., 2020).

Balance in cellular H_2_O_2_ levels is also maintained by enzymes of the ascorbate–glutathione cycle, such as APX, MDHAR, DHAR, and GR. APXs, as heme-containing peroxidases, detoxify H_2_O_2_ *via* the electron transfer from ascorbate to form monodehydroascorbate (MDHA) and H_2_O. The presence of nine putative *APX* genes has been described in the *Arabidopsis* genome; nevertheless, the *APX4* gene product is lacking H_2_O_2_ decomposing activity, and *APX7* is annotated as a pseudogene (Granlund et al., 2009). Cytosolic (APX1, APX2 and APX6), chloroplast (stromal sAPX and thylakoid tAPX), peroxisomal (APX3 and APX5) APXs have been recognized in *Arabidopsis* (Maruta et al., 2012, 2016), while sAPX is targeted also to mitochondria (Chew et al., 2003). Chloroplastic APX isozymes are involved in the water-water cycle, which decomposes H_2_O_2_ generated by O_2_^.−^ dismutation (Huang et al., 2019). They are therefore crucial for photoprotection (Murgia et al., 2004; Kangasjärvi et al., 2008; Maruta et al., 2010). Remarkably, the chloroplastic H_2_O_2_ detoxification turns to be inactive in plants depleted of cytosolic APX1 (Davletova et al., 2005a, Pnueli et al., 2003). APX1 is also involved in plant responses to heat and drought stress (Koussevitzky et al., 2008; Vanderauwera et al., 2011) and to wounding (Maruta et al., 2012). The importance of cytosolic APX2 was shown during high light, heat, salinity, and drought stresses using *apx2* and *apx1/apx2* mutants (Rossel et al., 2006; Suzuki et al., 2013). Additionally, both cytosolic APXs play important roles during cold stress (van Buer et al., 2016). Mutants in peroxisomal *APX3* do not display any phenotype upon salt treatment and exposure to low or high temperature (Narendra et al., 2006). Finally, APXs also play essential roles as enzymatic regulators of H_2_O_2_ signaling during plant development (Chen et al., 2014; Pandey et al., 2017; Chin et al., 2019).

The reverse reduction of MDHA to ascorbate, catalyzed by MDHAR, occurs in the presence of NAD(P)H as a reductant (Foyer and Noctor, 2011). Overall, five *Arabidopsis* genes encode six functional proteins of MDHARs (Obara et al., 2002). Cytosolic localization is confirmed for MDHAR2 and 3, whereas MDHAR1 has been found also in peroxisomes. MDHAR4 is located in peroxisomal membrane (Lisenbee et al., 2005; Eubel et al., 2008; Kaur and Hu, 2011). The *MDHAR6* gene is expressed in two splicing variants, producing two protein products localized either in mitochondria (MDHAR5) or chloroplasts (MDHAR6; Obara et al., 2002). The overexpression of *Arabidopsis MDHAR1* in tobacco leads to increased tolerance to ozone, salt, and osmotic stresses (Eltayeb et al., 2007). The overexpression of cytosolic *Acanthus ebracteatus* MDHAR in rice confers increased resistance to salt stress and higher germination rate and grain weight (Sultana et al., 2012). A study exploiting the genetic manipulation of *MDHAR4* suggests that it is implicated in plant germination, post-germination growth, and possibly in senescence (Eastmond, 2007). MDHAR2 and MDHAR5 play important roles during the interaction of *Arabidopsis* with plant growth-promoting endophyte *Piriformospora indica* (Vadassery et al., 2009).

Dehydroascorbate (DHA) is enzymatically reduced by DHAR by using glutathione as an electron donor, which is oxidized to glutathione disulfide. DHARs are soluble monomeric enzymes, and their thiol group participates in the catalyzed reaction. Three functional genes are present in the *Arabidopsis* genome. Their protein products are localized either in the cytosol (DHAR1, DHAR2) or chloroplasts (DHAR3; Rahantaniaina et al., 2017). Recently, their role in the regulation of ascorbate and glutathione homeostasis was described during plant developmental processes (reviewed in Ding et al., 2020). The overexpression of *DHAR1* protects *Arabidopsis* from methyl viologen-induced oxidative, high temperature, and high light stresses (Ushimaru et al., 2006; Wang et al., 2010; Noshi et al., 2017). DHAR2 has an antioxidant role in plant responses to ozone (Yoshida et al., 2006), drought, salt, and polyethylene glycol (Yin et al., 2010; Eltayeb et al., 2011), whereas DHAR3 is involved in the high light response (Noshi et al., 2016).

The pool of reduced glutathione consumed by DHAR activity is recovered by GR in a NADPH-dependent reaction, which is essential for glutathione homeostasis. Structurally, the GR protein contains a FAD-binding domain, a dimerization domain, and a NADPH-binding domain, crucial for proper enzymatic activity (Berkholz et al., 2008). Two isozymes were described in *Arabidopsis*, showing dual localization in the cytosol and peroxisomes for GR1 and in chloroplasts and mitochondria for GR2 (Kataya and Reumann, 2010; Marty et al., 2019). GR1 is involved in the tolerance of *Arabidopsis* to high light (Müller-Schüssele et al., 2020), heavy metals (Guo et al., 2016; Yin et al., 2017), and salt stress (Csiszár et al., 2018). GR2 is involved in methyl viologen-induced oxidative stress (Wang et al., 2019), chilling stress (Kornyeyev et al., 2003), and high light stress (Karpinski et al., 1997) but also in developmental processes such as root growth, root apical meristem maintenance (Yu et al., 2013), embryo development (Marty et al., 2019), and seed germination (Sumugat et al., 2010). In addition, the knockout mutation of *GR3* confers salt stress sensitivity in rice (Wu et al., 2015).

## Regulation of Antioxidant Enzymes

Plants can percept, transduce, and then translate the ROS signal into appropriate cellular responses. The key consequence of ROS accumulation is the modification of the potential signaling targets [e.g., kinases, transcription factors (TFs), and stress response-related proteins] by their oxidizing properties. ROS can modulate signaling through their capability to affect the protein redox status *via* the oxidation of methionine residues and thiol groups of cysteines. This leads to the activation/deactivation, structure alteration, and loss-/gain-of-function of ROS targets (Waszczak et al., 2015). Redox-related processes are strictly regulated by such proteins as thio- and glutaredoxins, which can undergo reversible oxidation/reduction and can be activated/inactivated in response to the cellular redox state (Waszczak et al., 2015, 2018). Recently, a redox-based sensing mechanism was introduced for H_2_O_2_, including cell-surface H_2_O_2_ receptor capable of transducing signal from extracellularly produced ROS into intracellular signaling cascades (Wu et al., 2020). HYDROGEN-PEROXIDE-INDUCED Ca^2+^ INCREASES 1 (HPCA1) is a membrane-spanning enzyme belonging to a protein family of leucine-rich repeat (LRR) receptor kinases, which percepts apoplastic H_2_O_2_ *via* the oxidation of two pairs of cysteine residues in its extracellular domain, leading to its autophosphorylation. This promotes the acceleration of Ca^2+^ influx through Ca^2+^-channels and the subsequent closure of stomata (Wu et al., 2020).

ROS can also cause carbonylation, a type of protein oxidation, where the carbonyl groups (aldehydes and ketones) are attached to protein side chains at proline, arginine, lysine and threonine residues (Yalcinkaya et al., 2019). Carbonylation alters protein stability and might enhance their susceptibility to proteolysis (Dalle-Donne et al., 2003; Suzuki et al., 2010; Ciacka et al., 2020).

Redox perturbations, driven by ROS produced in chloroplasts and mitochondria, are transduced by metabolic signals to activate rapid adaptive mechanisms by retrograde signaling (Chan et al., 2016; Cui et al., 2019). As of late, ROS were also introduced as mediators of retrograde signaling directed from the plastids to the nucleus. Thus, H_2_O_2_ generated in plastids is transported to the nucleus to activate the defense gene expression (Exposito-Rodriguez et al., 2017).

In addition, ROS can cross talk with other key secondary messengers, such as Ca^2+^ and RNS. Owing to their strong oxidation potential, ROS interact with such ubiquitous messengers as nitric oxide (NO), leading to the formation of RNS, including radical nitric oxide (NO^·^), nitric dioxide (${\text{NO}}_{2}^{\cdot }$), and nitrate radical (${\text{NO}}_{3}^{\cdot }$) as well as non-radical peroxynitrite (ONOO^−^), nitrosonium cation (NO^+^), nitroxyl anion (NO^−^), nitrous acid (HNO_2_), and other NO_x_ species involved in plant development, metabolism, (a)biotic stress responses, or stomatal closure (del Río, 2015; Lindermayr and Durner, 2015; Piterková et al., 2015; Farnese et al., 2016; Niu and Liao, 2016). The first evidence of NO and H_2_O_2_ interplay was related to cytotoxic effects during plant hypersensitive responses (Delledonne et al., 2001). Generally, the ROS cross talk with RNS is concentration-dependent and organelle- and even microcompartment-specific and might have beneficial or deleterious effects on plant cells (Kohli et al., 2019).

In general, an increased production of ROS caused by various environmental cues rapidly triggers antioxidant defense by multiple mechanisms, including retrograde signaling, transcriptional control, post-transcriptional regulation, post-translational redox modifications or phosphorylation, and protein–protein interactions.

### Redox Regulation

The cellular antioxidant capacity is tightly coupled with the maintenance of redox homeostasis by redox buffers such as ascorbate and glutathione (Karpinski et al., 1997; Foyer and Noctor, 2011). Both compounds may directly decompose ROS and are essential for preserving the ROS content at physiological levels. In addition, they serve as co-substrates for enzymes of the ascorbate–glutathione cycle. The high reduction state of both compounds is connected to enhanced plant tolerance to adverse stress conditions and increased antioxidant capacity (Foyer and Noctor, 2011). The flexibility and rapid response of antioxidant enzymes to changing external conditions are primarily controlled by the redox state of thiol groups in their amino acid sequences, regulated by thio-, peroxi-, and glutaredoxins or other oxidoreductases (Meyer et al., in press). One of them, NUCLEOREDOXIN 1 (NRX1), can target several important antioxidant enzymes, including CAT1, CAT2, and CAT3, GLUTATHIONE S-TRANSFERASE (GST), APX1, GLUTAREDOXIN FAMILY PROTEIN, and METHIONINE SULFOXIDE REDUCTASE (MSR) B2. The expression of antioxidant enzymes depends on the proper functioning of NRX1 that has an impact on plant oxidative stress tolerance (Kneeshaw et al., 2017). A similar proteomic elucidation of thioredoxin mitochondrial targets using affinity chromatography revealed that mitochondrial MSD1, thio- and peroxiredoxins, MSR isoforms, and GLUTATHIONE PEROXIDASE 6 act in a thioredoxin-dependent manner (Yoshida et al., 2013). Multiple antioxidant enzymes including chloroplastic CSD2, CAT3, MDHAR6, PEROXIREDOXIN TPx1, GST isoforms, and MSR-LIKE PROTEIN have also been detected as targets of THIOREDOXIN y1, a plastidic thioredoxin isoform in roots (Marchand et al., 2010). These data indicate that the redox regulation of antioxidant plant defense is quite complex and requires both spatial and temporal coordination.

### Mitogen-Activated Protein Kinase Signaling, Reactive Oxygen Species, and Antioxidants

#### Reactive Oxygen Species-Induced Mitogen-Activated Protein Kinase Signaling Pathways

A very significant feature of ROS is their capability to activate MAPKs, representing key signal transduction proteins triggered by a plethora of environmental and developmental factors (Colcombet and Hirt, 2008; Šamajová et al., 2013; Smékalová et al., 2014, Komis et al., 2018). MAPK signaling cascades consist of MAPKKKs (MAP3Ks), MAPKKs (MAP2Ks), and MAPKs, which are consecutively phosphorylated, leading to the activation/inactivation of a wide range of target proteins, including TFs (Liu and He, 2017). It is well-known that MAPK signaling pathways are activated by ROS accumulated during plant responses to either abiotic stresses or pathogen attack. So far, two kinds of MAP3Ks were identified to be activated by ROS, namely ARABIDOPSIS HOMOLOGS OF NUCLEUS AND PHRAGMOPLAST LOCALIZED KINASES (ANPs) and MITOGEN-ACTIVATED PROTEIN KINASE KINASE KINASE 1 (MAPKKK1 or MEKK1). ANPs are required for the plant immune response (Kovtun et al., 2000; Savatin et al., 2014), whereas the ROS-triggered signal is further transduced *via* MITOGEN-ACTIVATED PROTEIN KINASE 3 (MPK3) and MPK6 (Kovtun et al., 2000; Nakagami et al., 2006). The full activation of MPK3 and MPK6 is preconditioned by the presence of OXIDATIVE SIGNAL INDUCIBLE 1 kinase (OXI1), which is an essential component of this signal transduction pathway (Rentel et al., 2004). Another ROS-activated MAPK cascade consists of MEKK1, MKK1/2, and MPK4 (Nakagami et al., 2006; Pitzschke et al., 2009). This pathway is also important for basal plant defense against pathogen attack (Zhang et al., 2012). Moreover, a pathogen-induced oxidative burst activates the MPK7 downstream of MKK3 (MAP2K), thus triggering the expression of pathogenesis-related (PR) genes, independently of flagellin receptor FLAGELLIN SENSING 2 (Dóczi et al., 2007).

Furthermore, MPK1 and MPK2 are activated by oxidative stress and jasmonic acid (JA), ABA, and wounding (Ortiz-Masia et al., 2007). In turn, ABA and ROS-activated MPK9 and MPK12 act upstream of anion channels in guard cells, thus regulating stomatal closure (Jammes et al., 2009). MPK3 was found as another principal player in guard cell signaling *via* ABA and H_2_O_2_ perception in guard cells, which leads to stomatal closure (Gudesblat et al., 2007). Thus, MAPK signaling activated by ABA-induced ROS accumulation is generally implicated in stomatal movements (Danquah et al., 2014; Sierla et al., 2016).

MAPKs also respond to ROS produced in chloroplasts and mitochondria. In this respect, MPK6 is implicated in chloroplast to nucleus-directed retrograde signaling upon intense light exposure. The activation of MPK6 under such conditions is preceded by the export of Calvin–Benson cycle intermediate dihydroxyacetone phosphate (DHAP) from chloroplasts to the cytosol. These events lead to the rapid (within several minutes) expression of *APETALA2/ETHYLENE-RESPONSIVE ELEMENT BINDING FACTOR (AP2/ERF)* TFs and other downstream genes, such as *CHLOROPLAST PROTEIN KINASE LIKE* (*ChlPK-like*), *CHITINASE FAMILY PROTEIN* (*CHFP*), *HEAT SHOCK PROTEIN 20 LIKE* (*HSP20-like*), and *PR1* (Vogel et al., 2014). Similarly, MPK4 orchestrates plastid retrograde signaling in a salicylic acid-dependent manner (Gawroński et al., 2014). Mitochondrial ROS production induced by oxygen deprivation activates MPK6 and subsequent retrograde signaling toward the nucleus, leading to transcriptional reprogramming and triggering plant defense mechanisms (Chang et al., 2012).

Several studies report on the regulation of MAPKs by direct interactions with ROS. Waszczak et al. (2014) identified MPK2, MPK4, and MPK7 as capable of being sulfenylated in an H_2_O_2_-dependent manner. Another example pertains to *Brassica napus* BnMPK4, an ortholog of *Arabidopsis* MPK4, activated by H_2_O_2_ and undergoes aggregation upon the H_2_O_2_-dependent oxidation of the Cys232 residue (Zhang T. et al., 2015). Thus, it is obvious that MAPKs could be modified on cysteine residues by direct oxidation, affecting their stability, aggregation, and probably protein–protein interactions. It is noteworthy that the redox regulation of MAPKs may lead either to their activation or inactivation. The oxidation of kinase amino acid residues was reported to interfere with ATP binding and cause inactivation (Diao et al., 2010) but may also lead to their super-activation state (Corcoran and Cotter, 2013).

#### Regulation of Antioxidant Enzymes by Mitogen-Activated Protein Kinase Signaling

MAPKs appear as essential regulators of antioxidant defense, as their genetic modifications can alter the expression profile of many antioxidant enzymes under diverse environmental conditions.

Under high light intensity (Xing et al., 2013) and hypersalinity (Xing et al., 2015), the expression of *Arabidopsis* SODs is regulated by MKK5 (MAP2K). The expression of *CSD1* and *CSD2* increases under enhanced light exposure. Interestingly, the transcript levels of both *CSD* genes remain unchanged under these conditions in a transgenic *Arabidopsis* line with downregulated *MKK5*, which is hypersensitive to high light. In contrast, a transgenic *Arabidopsis* line overexpressing *MKK5* is resistant to high light stress and shows the increased activity of both CSDs. Moreover, the downregulation of *MKK5* negatively affects the activation of MPK3 and MPK6 under these conditions, thereby implying that these two MAPKs act downstream of MKK5 (Xing et al., 2013). It is worth mentioning that MKK5, acting downstream of MEKK1 and upstream of MPK6, is also essential for the expression of chloroplastic *FSD2* and *FSD3* during salt stress (Xing et al., 2015). In addition, MKK1 mediates the transcriptional activation of *CAT1*, but not of *CAT2* and *CAT3*, during salt stress, and after drought and ABA treatments (Xing et al., 2007). CAT1 is important for the regulation of ABA-mediated H_2_O_2_ production, whereas its expression is activated by MPK6 operating downstream of MKK1 (Xing et al., 2008).

A MAPK cascade activated by ROS includes ANPs, MPK3, and MPK6 (Kovtun et al., 2000). A shotgun proteomic analysis of *Arabidopsis anp2/anp3* double mutant revealed the overabundance and/or increased activity of several proteins important for plant antioxidative defense, including FSD1, MSD1, and enzymes of the ascorbate–glutathione cycle, such as APX and DHAR. Consequently, this double mutant showed enhanced resistance to the methyl viologen-induced oxidative stress. Thus, it might be concluded that ANPs negatively regulate *Arabidopsis* tolerance to oxidative stress (Takáč et al., 2014). ANPs are likely master regulators of plant antioxidant defense. Contrarily, they also confer resistance to *Arabidopsis* against necrotrophic fungus *Botrytis cinerea* (Savatin et al., 2014), suggesting the functional divergence of ANPs in *Arabidopsis*.

The genetic modification of the MEKK1-MKK1/MKK2-MPK4 signaling pathway also deregulates the expression of several antioxidant enzymes. Notably, *Arabidopsis* mutants in genes encoding individual constituents of this cascade show diverse patterns of *CSD1, APX1, GR, CAT1*, and *CAT2* expression. The upregulation of *CAT1* and *CAT3* in *mekk1* and *mkk1/mkk2* mutants, but not in the *mpk4* mutant points, to the complexity of MAPK signaling toward antioxidant genes suggests possible cross talk of diverse MAPK signaling pathways (Pitzschke et al., 2009). According to another study, MPK4 is required for the homeostasis of ROS scavenging proteins in a salicylic acid-dependent manner (Gawroński et al., 2014). Apart from its signaling role during pathogen defense, salicylic acid positively regulates the expression of diverse abiotic stress-related proteins, including antioxidant enzymes, thus contributing to plant stress tolerance (Horváth et al., 2007; Khan et al., 2015). The double mutant in *MPK4* and *ISOCHRISMATE SYNTHASE 1*, encoding an enzyme involved in the synthesis of salicylic acid, results in the deregulated expression of *FSDs, CSD2, GR2*, and *APXs*. Therefore, the cross talk of salicylic acid and MAPK signaling is important for the expression of enzymes with antioxidant functions (Gawroński et al., 2014).

**Figure 1:**
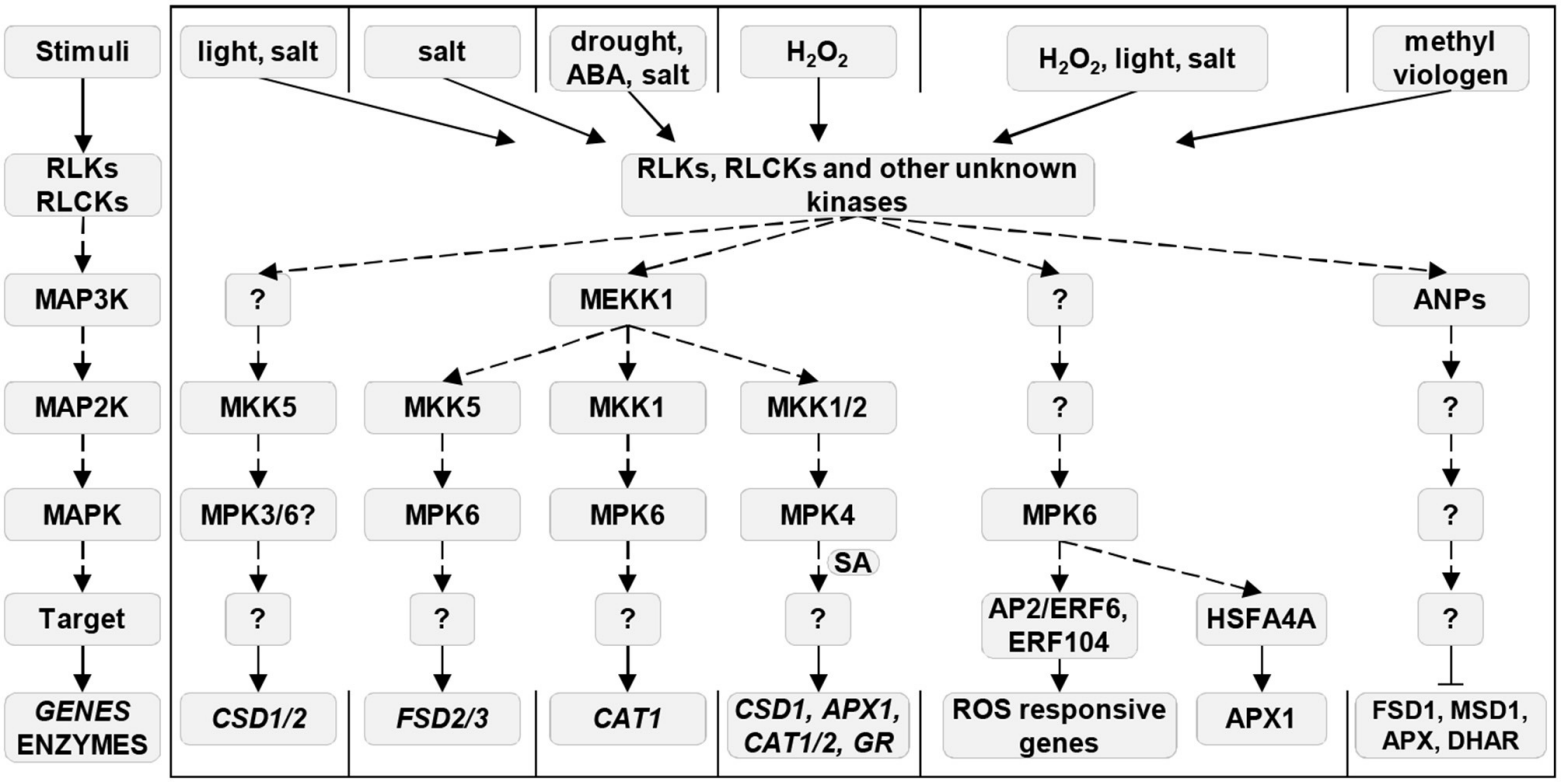
MAPK-dependent regulation of antioxidant enzymes during plant stress responses. Most left pathway shows the universal order of a MAP3K/MAP2K/MAPK cascade. Solid arrows indicate induction, dashed arrows show phosphorylation, and ⊥ indicates negative regulation. Question mark means an unknown component of the pathway. ABA, abscisic acid; ANPs, Arabidopsis nucleus- and phragmoplast-localized kinases; AP2/ERF6, Apetala2/Ethylene-responsive element binding factor 6; APX, ascorbate peroxidase; CAT, catalase; CSD, Cu/Zn superoxide dismutase; DHAR, dehydroascorbate reductase; ERF104, Ethylene-responsive element binding factor 104; FSD, Fe superoxide dismutase; GR, glutathione reductase; HSFA4A, Heat shock transcription factor A4A; MAPK, mitogen-activated protein kinase; MAP2K, mitogen-activated protein kinase kinase; MAP3K, mitogen-activated protein kinase kinase kinase; MSD, Mn superoxide dismutase; RLCKs, receptor-like cytoplasmic kinases; RLKs, receptor-like kinases; ROS, reactive oxygen species; SA, salicylic acid.

Thus, MEKK1 appears as an important regulator of antioxidant defense, capable of activation of MKK1/2 and MKK5 and subsequently MPK3/6 and MPK4. According to the current knowledge, MAPKs acting downstream of MEKK1 (MKK1/2/5; MPK3/4/6) may serve as proteins providing signal specificity toward individual antioxidant enzymes (Figure 1).

The MAPK-mediated transcriptional remodeling of oxidative stress-related genes occurs *via* the phosphorylation of TFs. For example, a TF called MYB44 (Persak and Pitzschke, 2013, 2014) and HEAT SHOCK TRANSCRIPTION FACTOR A4A (HSFA4A; Pérez-Salamó et al., 2014) are phosphorylated by MPK3 or MPK6. Heat shock TFs are known to play important roles during plant responses to several abiotic stresses. Thus, the overexpression of *HSFA4A* reduces the H_2_O_2_ content and lipid peroxidation, whereas it increases the activity of APX1 after salt treatment. Moreover, it leads to transcriptional changes in a large set of genes responsive to oxidative stress. The same study also confirmed that HSFA4A is involved in the transcriptional activation of genes encoding other TFs, such as *WRKY30, ZAT12, CRK13, HSP17.6A, ZAT6*, and *CTP1*, which are known to play essential roles in plant responses to biotic and abiotic stresses (Pérez-Salamó et al., 2014). Furthermore, MPK6 phosphorylates AP2/ERF6 (Wang et al., 2013a; Vogel et al., 2014) and AP2/ERF104 (Bethke et al., 2009) during oxidative stress. After activation, ERF6 specifically binds to the ROS-responsive cis-acting element 7 (ROSE7/GCC box), thus inducing the expression of ROS-responsive genes under intense illumination. ROSE are sorted into seven groups according to their *cis*-acting motives and core sequences showing different responses to stress conditions (Wang et al., 2013a).

**Figure 2:**
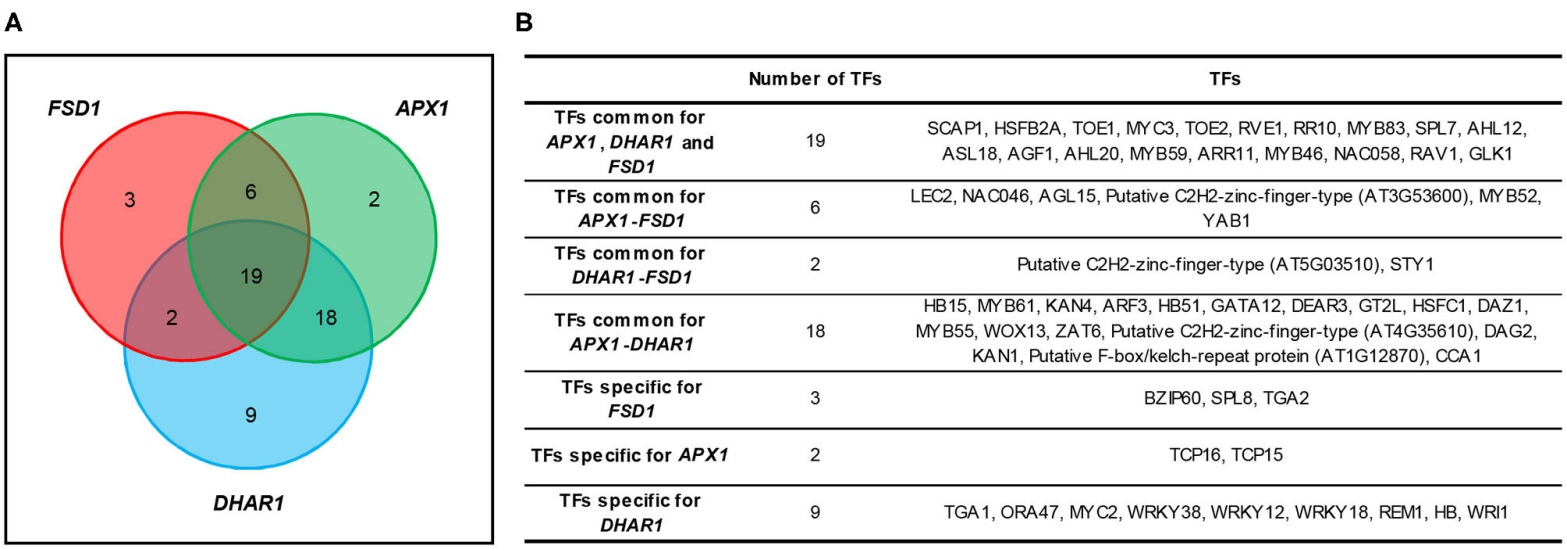
Graphical **(A)** and tabular **(B)** overview of common transcription factors (TFs) predicted to regulate the expression of *FSD1, APX1*, and *DHAR1* under the regulation of mitogen-activated protein kinases.

Thus, MAPKs can activate an array of TFs controlling the expression of antioxidant enzymes. Modern bioinformatics provides an opportunity to broaden the list of potential TF-regulating antioxidants, which show altered expression (transcriptomics) or abundance (proteomics) in transgenic lines with a modified or missing expression of particular *MAPK* genes. This may be carried out by integrating four different parameters: (1) the presence of *cis*-element(s) in the promoter sequence of the gene encoding the target antioxidant enzyme (predicted by AthaMap; Hehl et al., 2016), (2) TFs co-expressed with target antioxidants and MAPKs (determined by ATTED II; Obayashi et al., 2018), (3) TFs containing a MAPK-specific phosphorylation site [S(p)P or S(p)T; evaluated by PhosPhat 4.0 and GPS 3.0; Xue et al., 2005; Zulawski et al., 2013], and (4) the presence of a MAPK-specific docking site in the amino acid sequence of the TF (evaluated by ELM; Kumar et al., 2020). As an example, we provide a list of TFs potentially responsible for the expression of *FSD1, DHAR1*, and *APX1* under the regulation of MPK3, MPK4, and MPK6 (Figure 2; Supplementary Tables 1–3). Previously, these three enzymes showed significant changes in their abundance in *mpk4* (Takáč et al., 2016b) and *anp2/anp3* mutants (Takáč et al., 2014). Some predicted TFs have already been reported as regulators of antioxidant enzymes, including SPL7 regulating *FSD1*. The data set also includes HSFB2A, which has been predicted as a general TF for all three examined enzymes (Supplementary Table 1).

Finally, it should be noted that MAPK signaling can also affect the expression of *NOXs*. MEK2 cascade phosphorylates WRKY TFs (WRKY7/WRKY8/WRKY9/WRKY11), binding to the *cis*-element of the *RBOHB* promoter sequence during the exposure of *Nicotiana benthamiana* to bacterial protein elicitor INF1 and effector R3a/AVR3a (Adachi et al., 2015). In *Zea mays*, the activation of ZmMAPK5 is related to the increased expression of *NOX* and apoplastic ROS production in response to brassinosteroid and ABA treatments (Lin et al., 2009; Zhang et al., 2010). Nevertheless, the precise mechanism controlling the homeostasis between MAPK-induced ROS production and decomposition remains unknown.

### Ca^2+^ Signaling, Reactive Oxygen Species, and Antioxidants

#### Cross Talk of Reactive Oxygen Species With Ca^2+^ Signaling

Cytosolic Ca^2+^ is an important secondary messenger functioning in intra- and extracellular signaling networks involved in abiotic stress responses and plant innate immunity (Schulz et al., 2013; Demidchik et al., 2018; Tian et al., 2020). The rapidly fluctuating cytosolic content of Ca^2+^ is regulated by environmental cues, which activate Ca^2+^ channels, ion pumps, or plasma membrane- or organelle-membrane-embedded Ca^2+^ transporters (Chmielowska-Bak et al., 2014; Tian et al., 2020), as well as by Ca^2+^-binding proteins, such as calmodulin, calcineurin B-like proteins (CBL), and various Ca^2+^-dependent protein kinases (CPKs or CDPKs; Gilroy et al., 2014; Zhang et al., 2018). Plant- and protozoan-specific Ser-/Thr-CDPKs are key players of stress-mediated signaling networks, represented by a large number of members in the multigene family in diverse plant species [34 in *Arabidopsis*, 29 in rice, 20 in wheat, and 30 in poplar and *Brachypodium distachyon* (Zhang et al., 2018; reviewed in Atif et al., 2019; Wen et al., 2020)].

The interplay between Ca^2+^ and ROS is mutual because the cytosolic Ca^2+^ content is regulated by ROS, and *vice versa*, Ca^2+^ is crucial for ROS production (Choi et al., 2014; Gaupels et al., 2017). Therefore, Ca^2+^-dependent ROS signaling through NOXs as key signaling hubs and ROS-dependent Ca^2+^ signaling through the direct regulation of Ca^2+^ channels and sensors amplify each other during plant immune responses (reviewed by Marcec et al., 2019). The auto-propagating ROS wave generated by Ca^2+^-dependent NOX is directly linked to the Ca^2+^ wave during the systemic response of plants to pathogen infection, as CDPKs modulate the activity of RBOHD (reviewed by Gilroy et al., 2016). Both Ca^2+^ and ROS waves may be integrated *via* RBOHs, Ca^2+^ channel TWO-PORE CHANNEL 1, and CDPKs such as CPK/CBL-CIPKs (CBL-interacting protein kinases; Choi et al., 2014; Gilroy et al., 2016). Moreover, NOXs possess a special hydrophilic N-terminal Ca^2+^-binding site, the so called EF-hand motif, activated by Ca^2+^ (reviewed by Hu et al., 2020).

Under drought stress, ion channels in the plasma membrane are activated by ROS (Pei et al., 2000; Dodd et al., 2010), whereas H_2_O_2_ can induce the channel activity of *Arabidopsis* ANNEXIN 1 (AtANN1) and STELAR K^+^ OUTWARD RECTIFIER (SKOR; Richards et al., 2014; reviewed by Demidchik, 2018). In turn, CDPKs regulate the production of ROS by the phosphorylation of serine residues at the C-terminus of NOXs in a Ca^2+^-dependent manner (Kobayashi et al., 2007).

Moreover, *Arabidopsis* CPK5 and RBOHD are key components of a self-propagating activation circuit mediating cell-to-cell communication in plant immunity responses (Dubiella et al., 2013). Further, a plasma membrane anchored AtCPK27 is required for the plant response to salt stress, as its disruption causes oxidative burst and H_2_O_2_ accumulation in primary roots (Zhao et al., 2015). CPK4, CPK5, CPK6, and CPK11 regulate ROS production in *Arabidopsis*, possibly by the direct phosphorylation of RBOHB (Boudsocq et al., 2010). Finally, endoplasmic reticulum membrane-localized *B. napus* CPK2 interacts with RBOHD during cell death, accompanied by ROS accumulation (Wang et al., 2018).

Therefore, reciprocal ROS and Ca^2+^ signaling pathways orchestrate the production and accumulation of these secondary messengers and play vital roles in plant adaptation to adverse environmental conditions.

#### Modulation of Antioxidant Enzymes by Ca^2+^ Signaling

The impact of Ca^2+^ signaling on antioxidant enzymes was demonstrated by experiments with exogenous Ca^2+^ or the genetic manipulation of Ca^2+^ transporters. For example, CaCl_2_ enhances the tolerance of rice seedlings to arsenic stress by the reduction of ROS content and the stimulation of MDHAR, DHAR, CAT, GLUTATHIONE PEROXIDASE, and SOD (Rahman et al., 2015). Ca^2+^-dependent ATPases are Ca^2+^ transporters involved in multiple stress signaling pathways, physiological processes such as stomatal closure and programmed cell death, and ROS homeostasis (reviewed by Marcec et al., 2019). Furthermore, OsACA6, a Ca^2+^-ATPase from rice, can modulate ROS levels under salinity and drought stresses by the upregulation of CAT, APX, and GR activities, whereas plants overexpressing *OsACA6* show enhanced tolerance to these abiotic stress factors (Huda et al., 2013).

It is known that CDPKs/CPKs are crucial for the regulation of both ROS generation and metabolism under both abiotic and biotic stress conditions and are closely related to enhanced antioxidant enzyme activities in plants, e.g., under the attack of herbivores or microbial pathogens (Romeis and Herde, 2014; Marcec et al., 2019). For instance, AtCPK8 is a component of the ABA- and Ca^2+^-mediated signaling pathway, which phosphorylates and activates CAT3 at Ser261 in stomatal guard cells during drought stress (Zou et al., 2015). The CPK-mediated regulation of CAT activity was reported under salt stress conditions, namely CPK12 controls ion homeostasis and ROS accumulation *via* CAT, APX, and SOD activities, thereby impacting *Arabidopsis* salt stress tolerance (Zhang et al., 2018). CPKs also affect other components of the ROS-decomposing machinery. Thus, OsCPK12 from rice enhances salt stress tolerance through decreasing the ROS content, owing to the elevated expression of *OsAPX2, OsAPX8*, and reduced expression of NOX called *OsBOHI* but, at the same time, negatively modulates blast disease resistance (Asano et al., 2012). Ca^2+^-regulated expression of *APX* was also observed in maize, as Ca^2+^ activation of ZmCCaMK (Ca^2+^/calmodulin-dependent protein kinase from *Z. mays*) is required for the expression and activation of *APX2* (and also *SOD4*) in response to brassinosteroids (Yan et al., 2015).

**Figure 3:**
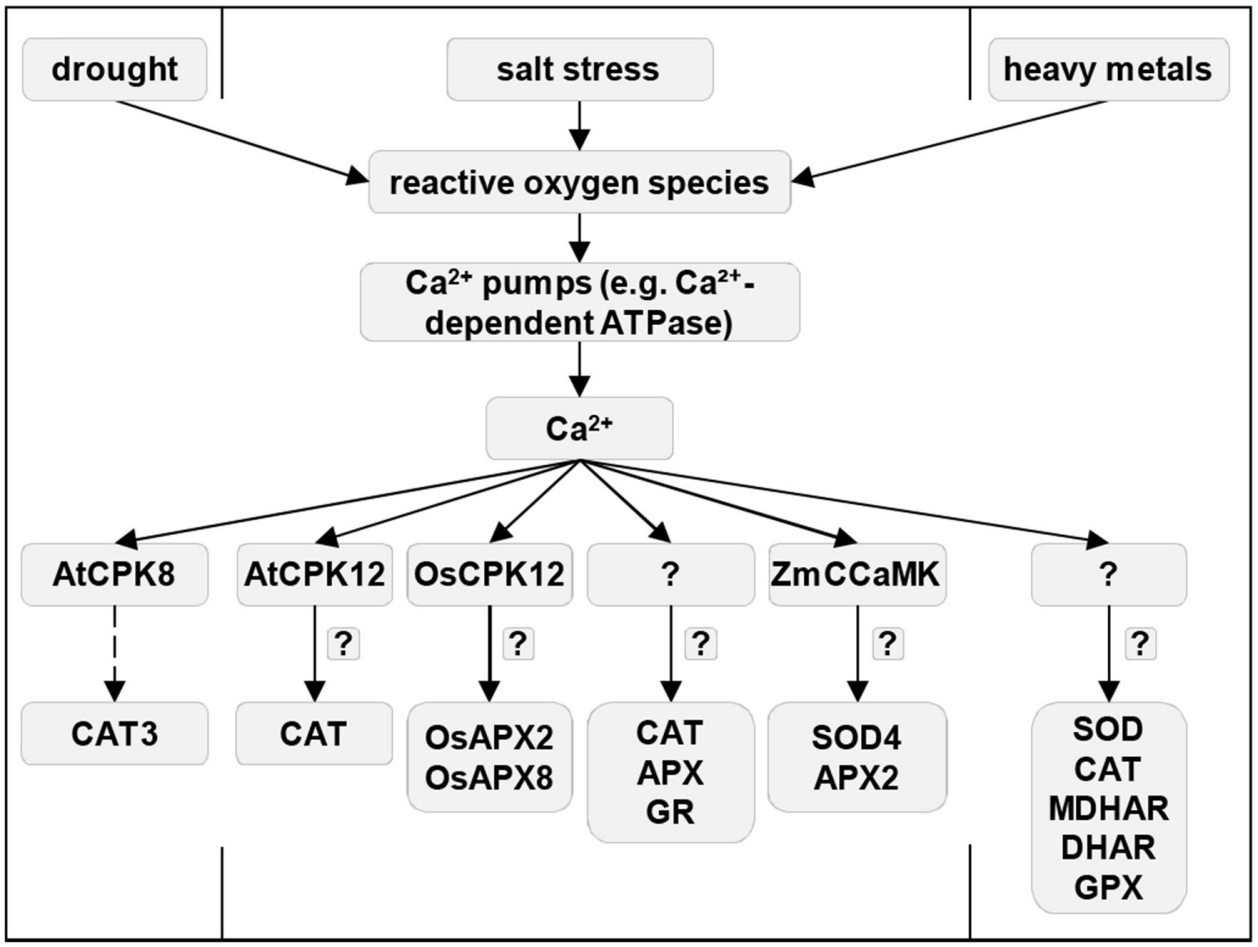
Mechanisms of Ca^2+^-mediated regulation of antioxidant enzymes during stress in plants. Solid arrows indicate induction, and dashed arrows show phosphorylation. Question mark means an unknown regulation. APX, ascorbate peroxidases; CAT, catalase; CPK, Ca^2+^-dependent protein kinases; DHAR, dehydroascorbate reductase; GPX, glutathione peroxidase; GR, glutathione reductase; MDHAR, monodehydroascorbate reductase; SOD, superoxide dismutase; ZmCCaM, Ca^2+^/calmodulin-dependent protein kinase from *Zea mays*.

Hence, Ca^2+^ homeostasis and subsequent Ca^2+^ signal transduction *via* CDPKs (Figure 3) play an inevitable role in the regulation of antioxidant protection machinery by enhancing plant plasticity and resistance to environmental challenges.

### Reactive Nitrogen Species-Mediated Regulation of Antioxidant Enzymes

RNS interplay with ROS-signaling pathway by both direct and indirect modulation of antioxidant enzymes activity (Lindermayr and Durner, 2015; Farnese et al., 2016). Thus, exogenous NO donor sodium nitroprusside (SNP) alleviates the detrimental effects of arsenic stress by stimulating the SOD, CAT, GST, and APX activities (Shukla and Singh, 2015). Potentiated action of SNP and silicon (Si) significantly increased the activities of SOD, guaiacol peroxidase, APX, GR, and GST in arsenic-stressed *B. juncea* plants (Ahmad et al., 2020). SNP also alleviates cadmium (Cd^2+^)-induced oxidative damage in *O. sativa* by the pronounced enhancement of the SOD, APX, guaiacol peroxidase, and CAT activities (He et al., 2014). A similar mechanism of the protective effects of SNP under nickel stress by upregulating the transcript levels of CAT, guaiacol peroxidase, APX, GR, and SOD genes was described (Rizwan et al., 2018). Both NO and H_2_O_2_ are involved in the regulation of ascorbate and glutathione metabolism by JA in *Agropyron cristatum* leaves, as JA stimulates the production of both secondary messengers and enhances the activities of APX, GR, MDHAR, DHAR, and enzymes of ascorbate biosynthesis (Shan and Yang, 2017). The link between NO and glutathione exists as well, as SNP modulates glutathione synthesis in *Medicago truncatula* roots by upregulation of *GAMMA-GLUTAMYLCYSTEINE SYNTHETASE* and *GLUTATHIONE SYNTHETASE* but not *HOMOGLUTATHIONE SYNTHETASE* (Innocenti et al., 2007).

Covalent loss- and/or gain-of-function NO-induced post-translational modifications (PTM) of antioxidant enzymes such as S-nitrosylation and tyrosine nitration reciprocally regulate ROS homeostasis, thereby preserving the balance between ROS production and scavenging in plant cells under natural and stress conditions (Yang et al., 2015; Romero-Puertas and Sandalio, 2016; Kohli et al., 2019). Thus, S-nitrosylation at Cys32 increases the activity of cytosolic APX1 under salinity stress (Begara-Morales et al., 2014), enhances the plant resistance to methyl viologen, but negatively modulates the plant immune response triggered by flagellin elicitor peptide flg22 (Yang et al., 2015). ROS reduces the degree of S-nitrosylation through inhibition of S-nitrosoglutathione reductase (GSNOR), a Zn^2+^-dependent class III alcohol dehydrogenase enzyme controlling the pool of S-nitrosoglutathione (GSNO)-the storage and long-distance transport NO form. This mechanism leads to higher glutathione levels, transcripts, and activities of glutathione-dependent antioxidant enzymes (Kovacs et al., 2016). This PTM at Cys230 inhibits CAT1 activity in response to Cd^2+^ in pea leaves (Ortega-Galisteo et al., 2012). In turn, another NO-induced PTM, namely ONOO^−^-induced tyrosine nitration, inhibits the activity of APX (Begara-Morales et al., 2014), MDHAR (Begara-Morales et al., 2015), CAT (Chaki et al., 2015), MSD1, peroxisomal CSD3, and chloroplastic FSD3 (Holzmeister et al., 2015). It is noteworthy that CAT activity is affected not only by S-nitrosylation but also by H_2_S-induced persulfidation (Palma et al., 2020).

GSNOR is apparently a significant convergence point between ROS- and RNS-signaling, as ROS inhibits GSNOR activity by oxidation or the Zn^2+^-release from the GSNOR structure (Tichá et al., 2017; Lindermayr, 2018). The activity and stability of GSNOR1 are also regulated by ROG1 (REPRESSOR OF GSNOR1)-mediated transnitrosylation at Cys10, which, by this mechanism, affects NO-based redox signaling in plants. Surprisingly, ROG1 is identical to CAT3 (Chen et al., 2020), assigning unexpected roles to this antioxidant enzyme. Last but not least, ROS and RNS cooperatively regulate MAPK signaling in a broad range of abiotic stresses, such as heavy metal exposure as well as drought and osmotic stress (reviewed by Farnese et al., 2016).

**Figure 4:**
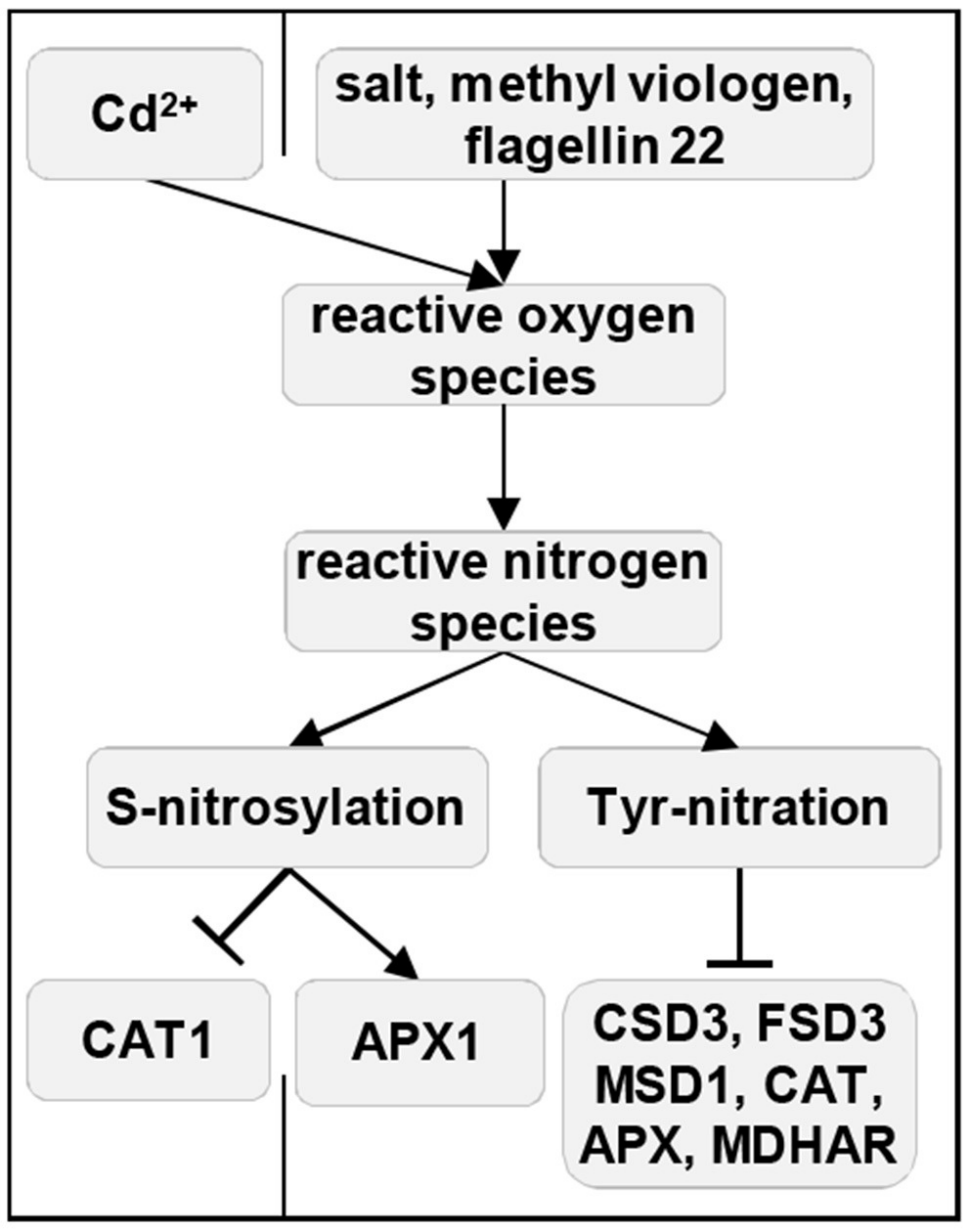
Reactive nitrogen species-mediated direct regulation of antioxidant enzymes during plant stress responses. Solid arrows indicate induction, and ⊥ indicates negative regulation. APX, ascorbate peroxidase; CAT, catalase; FSD, Fe superoxide dismutase; MDHAR, monodehydroacorbate reductase; MSD, Mn superoxide dismutase.

In summary, the expression and activity of antioxidant enzymes indispensably depend on their RNS mediated PTMs (Figure 4).

### Transcriptional and Post-transcriptional Regulation of Antioxidant Enzymes

Antioxidant enzymes are also regulated at the level of transcription, which is mediated by diverse TFs, not necessarily acting downstream of MAPKs. They are rapidly activated by redox perturbations in photosynthetic electron transport during retrograde signaling from plastid to nucleus encompassing metabolites (e.g., phosphoadenosines, tetrapyrroles, carotenoid oxidation products, carbohydrate metabolites, and isoprenoid precursors) and ROS (Chan et al., 2016; Exposito-Rodriguez et al., 2017). The rapid activation of MAPK cascades conditions this signaling process. Furthermore, TFs alone might be controlled by redox modifications (Dietz, 2014).

Compelling evidence suggests the transcriptional remodeling of ROS-responsive genes (reviewed in He et al., 2018; Li et al., 2018). Gadjev et al. (2006) performed a microarray transcriptomic analysis focused on the expression of exogenous ROS-induced TFs. More than 500 annotated *Arabidopsis* TFs showed a distinct expression pattern upon ROS, and some of them displayed the ability to modulate the expression of antioxidant enzymes (Gadjev et al., 2006). TF families most often connected to the transcriptional control of antioxidants in response to ROS include WRKY, Zinc finger (Znf), NAC, AP2/ERF, DREB, MYB, or bZIP (Khedia et al., 2019).

In general, numerous reports demonstrate the altered expression of antioxidant enzymes in transgenic or mutant plants with modified TF expression. Contrarily, only a few studies demonstrate the direct binding of TFs to the promoter sequences of antioxidant enzymes. It is known that *FSD1* and *CSD*s are transcriptionally orchestrated *via* a TF called SQUAMOSA PROMOTER BINDING PROTEIN-LIKE 7 (SPL7; Yamasaki et al., 2007, 2009; Garcia-Molina et al., 2014) in a Cu-dependent manner. Under copper deficiency, SPL7 binds to an SPL-specific SBP (SQUAMOSA PROMOTER BINDING PROTEIN DOMAIN) promoter sequence and induces the expression of *FSD1*, leading to increased abundance and activity of the FSD1 enzyme. At the same time, *CSD1, CSD2*, and COPPER CHAPERONE FOR SOD (*CCS*) are post-transcriptionally downregulated by miR398, which is induced by the binding of SPL7 to its promoter sequence (Cohu et al., 2009; Yamasaki et al., 2009). Therefore, SPL7 is an important modulator of copper balance *via* Cu-responsive proteins and miRNAs (reviewed by Pilon et al., 2011; Pilon, 2017; Araki et al., 2018). In turn, miR398 suppresses the mRNA of *CSD1* and *CSD2* after treatment with high sucrose content in the culture medium (Dugas and Bartel, 2008). Importantly, miR398 levels are downregulated to allow the post-transcriptional *CSD1* and *CSD2* mRNA accumulation leading to elevated (oxidative) stress tolerance (Sunkar et al., 2006; Khraiwesh et al., 2012). SPL7 is also implicated in the circadian regulation of *FSD1* expression (Perea-García et al., 2016). Further, *CSD1* and *CSD2* expression is also regulated by miR408 (Ma et al., 2015), illustrating that the post-transcriptional control of *SOD* expression is quite complex.

The equilibrium between *CSDs* and *FSD1* is influenced by *ZAT12* (Znf protein) because its overexpression leads to the increased expression of *CSD1, CSD2*, and *CCS*, while *FSD1* is downregulated (Davletova et al., 2005b). The induction of *ZAT12* expression is reported as an abiotic stress marker during high light exposure, low temperature, oxidative stress (triggered by H_2_O_2_ and methyl viologen), and osmotic and salinity stress (Kreps et al., 2002; Rizhsky et al., 2004a; Davletova et al., 2005b; Vogel et al., 2005; Xu et al., 2017). Furthermore, the expression of *FSD1, APX1*, and *APX2* depends on ZAT10 (Mittler et al., 2006) and RELATED TO APETALA-2.6L (RAP2.6L; Liu et al., 2012), while this positive regulation determines the resistance of *Arabidopsis* to abiotic stresses.

MYB49 has been reported to be an important TF for plant responses to drought, salt, and heavy metal stresses (Cui et al., 2018; Zhang et al., 2019; Zhang P. et al., 2020). In this respect, a transgenic tomato line overexpressing *MYB49* showed higher resistance to drought and salt stresses and increased its SOD and peroxidase activity (Cui et al., 2018). Additionally, an *Arabidopsis* transgenic line overexpressing functional repressor of MYB49, a chimeric AtMYB49-SRDX called SRDX49, showed downregulation of *CSD2* and several peroxidases (Zhang P. et al., 2020). Nevertheless, the binding of the TFs of ZAT, RAP, or MYB families to the promoter sequence of SODs has not been confirmed yet.

The expression patterns of *CATs* are closely correlated with plant senescence, and they contribute to the redox control of this process (Zimmermann et al., 2006; Mhamdi et al., 2012). The transcriptional regulation of *CATs* is complex and requires several TFs. *CATs* are directly controlled by WRKY53, which works downstream of redox-sensitive regulatory factor WRKY25 during senescence and oxidative stress response (Miao et al., 2004; Doll et al., 2020). *Arabidopsis wrky25/cat2* double mutant exhibit upregulated H_2_O_2_ levels in comparison with the wild-type or *cat2* single mutant (Doll et al., 2020). This ROS inducible WRKY25-WRKY53-CAT signaling hub can cross talk with MEKK1, which interacts with and phosphorylates WRKY53 (Miao et al., 2007). In addition, the *CAT2* promoter interacts with WRKY75, leading to *CAT2* downregulation depending on plant age, senescence, salicylic acid, H_2_O_2_, and multiple plant hormones (Guo et al., 2017). The *CAT3* expression is directly induced by two isoforms of AP2/ERF4 family TFs, having a different impact on plant senescence according to the alternative polyadenylation of pre-mRNAs. The age-dependent expression of *CAT3* and plant senescence are controlled by the expression ratio of *ERF4-A* (inducer) and *ERF4-R* (repressor) isoforms (Riester et al., 2019). Recently, MYC2 was experimentally approved to bind to the promoter of *Arabidopsis CAT2*, leading to its downregulation during leaf senescence in a JA-dependent manner. Such downregulation promotes H_2_O_2_ accumulation and the activation of senescence-associated genes (Zhang Y. et al., 2020). Finally, *CAT2* expression is also regulated by CDF4 (CYCLING DOF FACTOR 4) during senescence (Xu et al., 2020).

The ascorbate–glutathione cycle represents a very sensitive and efficient system for H_2_O_2_ decomposition, requiring not only strict redox control but also the synchronized expression of respective enzymes. APXs are major components of the ascorbate–glutathione cycle and comprise chloroplastic, peroxisomal, mitochondrial, and cytosolic isoforms, which efficiently eliminate excessive H_2_O_2_ by using ascorbate as an electron donor (Foyer and Noctor, 2011). The transcriptional control of *APXs* relies on multiple TFs, whereas RAP2s (also known as ERF-VIIs) exhibit the highest affinity toward both chloroplastic and cytosolic *APXs*. Chloroplastic stromal and thylakoid *APX* isoforms (Rudnik et al., 2017) and *2-Cys PEROXIREDOXIN A* (Shaikhali et al., 2008; Rudnik et al., 2017) are regulated by redox-sensitive RAP2.4a directly interacting with their promoters under photooxidative stress and increased ROS levels. Seven additional members of the RAP2.4 family also demonstrate an ability to bind to the promoters of the genes mentioned earlier and to fine-tune their expression (Rudnik et al., 2017). In addition, RADICAL-INDUCED CELL DEATH 1 (RCD1), a protein integrating mitochondrial and chloroplastic ROS signals with pleiotropic functions (Shapiguzov et al., 2019), interacts with RAP2.4 under mild and severe oxidative stress and promotes the activation of downstream genes (Hiltscher et al., 2014). The preference of RAP2 for *APXs* regulation has also been demonstrated for RAP2.6L because the overexpression of this TF causes the overexpression of cytosolic *APX1* and improves plant tolerance against waterlogging stress (Liu et al., 2012). Besides RAP2.6L, ZAT12, ZAT7, and WRKY25 are also required for the proper expression of cytosolic *APX1* during oxidative stress (H_2_O_2_, methyl viologen, heat shock, or wounding; Rizhsky et al., 2004b). *APX2* and *APX7* are expressed under the control of HSFA3, downstream of a TF named DEHYDRATION-RESPONSIVE ELEMENT-BINDING 2C (DREB2C), having an impact on plant resistance to oxidative stress (Hwang et al., 2012; Song et al., 2016). The induction of *APX2* transcription as a response to high light initiates photosynthetic redox homeostasis alterations occurring upon the nuclear accumulation and activation of HSFA1D (Jung et al., 2013). C2H2-type zinc finger protein ZFP36 is among the important *APX* regulators, as this TF binds to the promoter sequence and activates the *OsAPX1* gene in rice. This activation is enhanced by LATE EMBRYOGENESIS ABUNDANT 5 (OsLEA5), a protein interacting with ZFP36, which regulates *OsAPX1* expression during seed germination (Huang et al., 2018). In addition, ZFP36 was found to be important during rice responses to oxidative and abiotic stresses (Zhang et al., 2014). Another TF called ANAC089 has been reported as a negative regulator of stromal *APX* in *Arabidopsis* and can play different roles during plant acclimation to low, normal, and high light conditions. ANAC089 decreases the expression of stromal *APX* under highly reducing conditions induced by a DTT treatment (Klein et al., 2012).

Knowledge on the transcriptional control of *DHARs, MHARs*, and *GRs*, encoding enzymes implicated in ascorbate regeneration during the ascorbate–glutathione cycle, is limited. The mutant analysis of an AP2/ERF domain-containing TF showed the upregulation of *DHAR1* and the downregulation of *MDHAR3* in response to H_2_O_2_ treatment (Sewelam et al., 2013). Recently, R2R3-type MYB from *Pyrus betulaefolia* (PbrMYB5) was reported as TF important for the expression of *PbrDHAR2*. The genetic manipulation of PbrMYB5 in tobacco positively correlates with chilling when overexpressor lines show a higher expression of *NtDHAR2* and elevated levels of ascorbate (Xing et al., 2019). Finally, regulation by miRNA (PN-2013) interference was reported for wheat *MDHAR* (Feng et al., 2014).

### Phosphorylation of Antioxidant Enzymes

PTMs of proteins represent versatile, dynamic, and flexible regulatory mechanisms for the reprogramming of a wide range of cellular functions. For example, these processes ensure the fast and targeted activation of plant immune responses upon pathogen attacks. Generally, PTMs affect enzymatic activities, subcellular localization, protein interactions, and stability (reviewed in Withers and Dong, 2017; Ruiz-May et al., 2019; Vu et al., 2018;). Among PTMs, the reversible phosphorylation of proteins represents a driving force of signaling processes during plant development and stress challenges (Arsova et al., 2018). Several antioxidant proteins are modified by direct phosphorylation, mainly by proteomic approaches (Table 1).

**Table 1:** Phosphorylation sites experimentally found in antioxidant enzymes by mass spectrometry, retrieved from PhosPhat database.

**Protein**	**Description**	**Modified peptide**	**Treatment**	**Position**	**References**
AT2G28190	Copper/zinc superoxide dismutase 2	ALTVV(pS)AAK	Auxin	S62	Zhang et al., 2013
AT5G18100	Copper/zinc superoxide dismutase 3	GGHKLSK(pS)TGNAGSR	Isoxabene	S141	n.a.
AT3G10920	Manganese superoxide dismutase 1	NLAPS(pS)EGGGEPPK	Isoxabene	S114	n.a.
AT5G51100	Iron superoxide dismutase 2	EQEGTE(pT)EDEENPDDEVPEVYLD(pS)DIDVSEVD	Abscisic acid	S297_T280	Wang et al., 2013b
		EQEGTETEDEENPDDEVPEVYLD(pS)DIDVSEVD	Abscisic acid	S297	Wang et al., 2013b
		EQEG(pT)ETEDEENPDDEVPEVYLD(pS)DIDVSEVD	Abscisic acid	S297_T278	Wang et al., 2013b
		EQEGTE(pT)EDEENPDDEVPEV(pY)LDSDIDVSEVD	Abscisic acid	Y294_T280	Wang et al., 2013b
		EQEGTE(pT)EDEENPDDEVPEVYLDSDIDVSEVD	Abscisic acid	T280	Wang et al., 2013b
		EQEGTETEDEENPDDEVPEV(pY)LDSDIDVSEVD	Abscisic acid	Y294	Wang et al., 2013b
AT1G20630	Catalase 1	YPT(pT)PIV(C*)SGNR	Cell culture	T409	Sugiyama et al., 2008
AT4G35090	Catalase 2	LNVRP(pS)I		S491	Bhaskara et al., 2017
			Abscisic acid	S491	Umezawa et al., 2013
		TF(pT)PERQER	Ionizing radiation	T439	Roitinger et al., 2015
AT1G20620	Catalase 3	CAEKVP(pT)PTNSYTGIR	flg22	T408	Rayapuram et al., 2018
					Rayapuram et al., 2014
			End of day		Reiland et al., 2009
			flg22		Rayapuram et al., 2018
			flg22		Rayapuram et al., 2014
		CAEKVPTP(pT)NSYTGIR	flg22	T410	Rayapuram et al., 2014
			flg22		Rayapuram et al., 2018
		VP(pT)PTN(pS)YTGIR	Ionizing radiation	T408_S412	Roitinger et al., 2015
		GFFEVTHDISNL(pT)CADFLR	Nitrogen starvation/nitrate resupply	T85	Engelsberger and Schulze, 2012
		LNVRP(pS)I		S491	Bhaskara et al., 2017
			Abscisic acid		Umezawa et al., 2013
		(C*)AEKVPTPTNS(pY)TGIR	Abscisic acid	Y413	Wang et al., 2013b
		(C*)AEKVPTPTNSY(pT)GIR	Abscisic acid	T414	Wang et al., 2013b
AT4G08390	Stromal ascorbate peroxidase	VDASGPED(C*)PEEGRLPDAGPP(pS)PATHLR		S236	Nakagami et al., 2010
			Nitrate starvation/nitrate resupply	S236	Wang et al., 2012
			Abscisic acid	S236	Wang et al., 2013b
				S236	Van Leene et al., 2019
			Nitrate starvation/nitrate resupply	S236	Wang et al., 2013c
		VDASGPED(C*)PEEGRLPDAGPPSPA(pT)HLR	Abscisic acid	T239	Wang et al., 2013b
AT4G32320	Ascorbate peroxidase 6	FFEDF(pT)NA(pY)IK	Nitrogen starvation/nitrate resupply	T313_Y316	n.a.
AT1G77490	Thylakoidal ascorbate peroxidase	ELSD(pS)(oxM)(K*)(K*)	Ionizing radiation	S373	Roitinger et al., 2015
		(oxM)ISPK(C*)AA(pS)DAAQLISAK	flg22	S81	Mithoe et al., 2012
		LPDAGPP(pS)PADHLR	Ionizing radiation	S215	Roitinger et al., 2015
AT5G16710	Dehydroascorbate reductase 1	FQPST(pT)AGVLSASVSRAGFIKR	Abscisic acid	T11	Umezawa et al., 2013
AT1G75270	Dehydroascorbate reductase 2	(s)KDANDG(s)EKALVDELEALENHLK	Ethylene, ambient air		Li et al., 2009
AT3G52880	Monodehydroascorbate reductase 1	VVGAFMEGG(pS)GDENK		S400	Sugiyama et al., 2008
			Ionizing radiation	S400	Roitinger et al., 2015
				S400	Nakagami et al., 2010
				S400	Van Leene et al., 2019
		ARP(pS)AESLDELVK		S416	Nakagami et al., 2010
			None	S416	Reiland et al., 2011
			Nitrate starvation/nitrate resupply	S416	Wang et al., 2013c
			None	S416	Mayank et al., 2012
				S416	Reiland et al., 2009
				S416	Bhaskara et al., 2017
				S416	Van Leene et al., 2019
			flg22	S416	Rayapuram et al., 2018
				S416	Choudhary et al., 2015
			Abscisic acid	S416	Wang et al., 2013b
			Abscisic acid	S416	Umezawa et al., 2013
			Abscisic acid	S416	Xue et al., 2013
				S416	Sugiyama et al., 2008
			Ionizing radiation	S416	Roitinger et al., 2015
		ARP(s)AE(s)LDELVKQGI(s)FAAK			Reiland et al., 2009
		ARP(s)AE(s)LDELVKQGI(s)FAAK	None		Reiland et al., 2011
		ADLSAK(pS)LVSATGDVFK	Abscisic acid	S104	Umezawa et al., 2013
		ARPSAE(pS)LDELVK	Nitrate starvation/nitrate resupply	S419	Wang et al., 2013c
		GAD(pS)(K*)NILYLR	Ionizing radiation	S139	Roitinger et al., 2015
AT5G03630	Monodehydroascorbate reductase 2	AQP(pS)VESLEVLSK	flg22	S417	Rayapuram et al., 2018
			End of night	S417	Reiland et al., 2009
			flg22	S417	Rayapuram et al., 2018
			Ionizing radiation	S417	Roitinger et al., 2015
			flg22	S417	Rayapuram et al., 2014
			None	S417	Reiland et al., 2011
			flg22	S417	Rayapuram et al., 2018
			flg22	S417	Rayapuram et al., 2018
			End of night	S417	Reiland et al., 2009
			None	S417	Mayank et al., 2012
			Nitrate starvation/nitrate resupply	S417	Wang et al., 2013c
			Abscisic acid	S417	Wang et al., 2013b
				S417	Van Leene et al., 2019
				S417	Sugiyama et al., 2008
			None	S417	Reiland et al., 2011
			Abscisic acid	S417	Umezawa et al., 2013
			Ethylene	S417	Yang et al., 2013
			Abscisic acid, mannitol	S417	Xue et al., 2013
				S417	Choudhary et al., 2015
		AQP(pS)VE(pS)LEVLSK	flg22	S420_S417	Rayapuram et al., 2018
		VVGAFLEGG(pS)PEENNAIAK	Abscisic acid	S401	Wang et al., 2013b
AT3G09940	Monodehydroascorbate reductase 3	G(pT)VA(pT)GFSTNSDGEVTEVK	Epibrassinolide	T228_T231	Lin et al., 2015
AT3G27820	Monodehydroascorbate reductase 4	GTVLTSFEFD(pS)N(K*)(K*)	Ionizing radiation	S234	Roitinger et al., 2015
AT3G54660	Glutathione reductase 2	(pT)AAGV	drought stress	T561	Bhaskara et al., 2017

**Figure 5:**
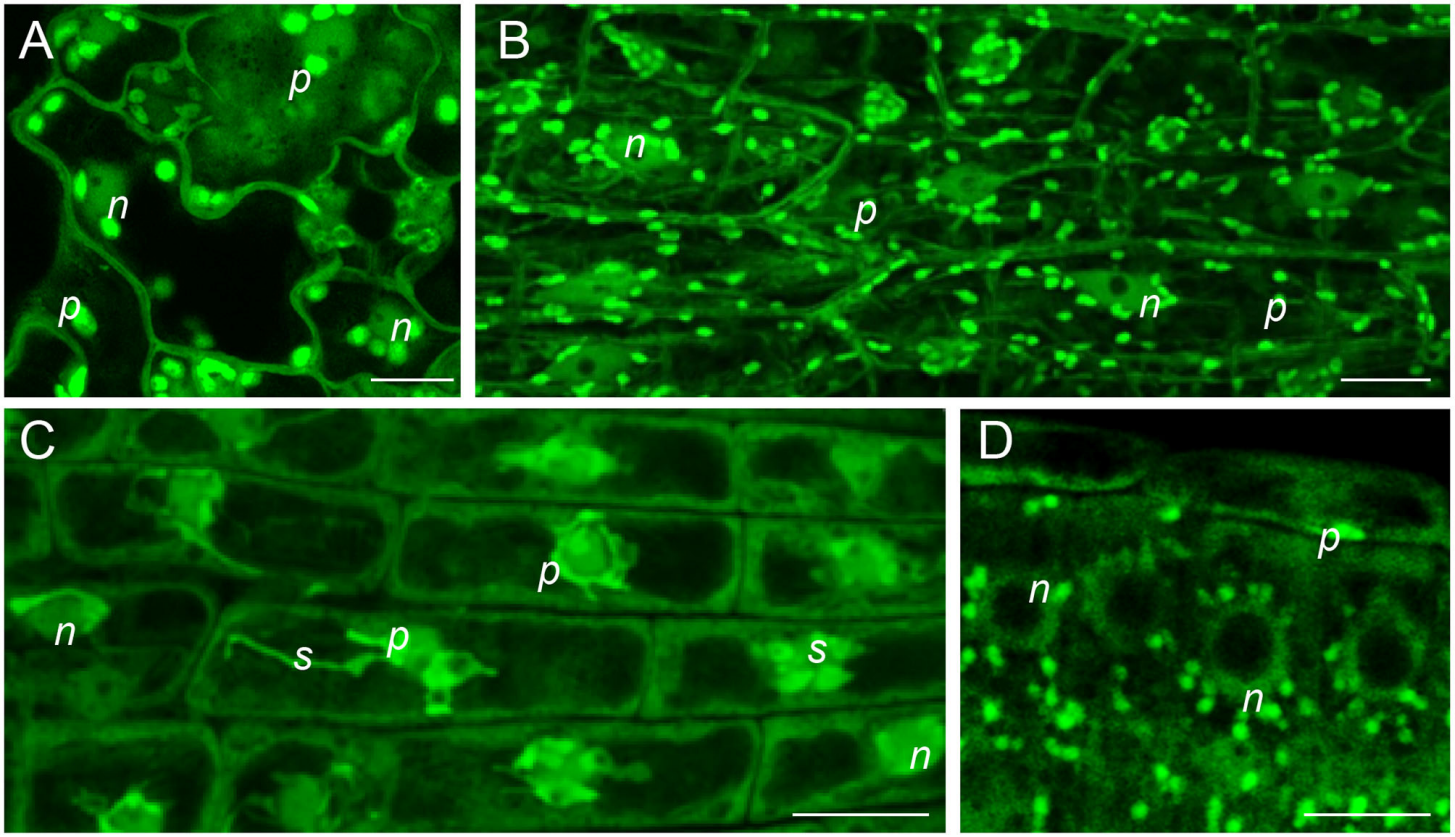
*In vivo* subcellular localization of FSD1-GFP in plastids, nuclei and cytoplasm of 4-day-old *Arabidopsis fsd1-1* mutant harboring *proFSD1::FSD1:GFP* construct (Dvořák et al., 2020) as revealed by confocal laser scanning microscope equipped with an Airyscan detector. **(A)** Pavement cells of leaf epidermis; **(B)** epidermal cells of hypocotyl; **(C)** lateral root cap cells; **(D)** root meristematic cells. n, nucleus; p, plastid; s, stromule. Scale bars: **(A**, **C**, **D)** 10 μm; **(B)** 20 μm.

Although the phosphorylation of SODs has been documented for human SOD1 (Fay et al., 2016; Tsang et al., 2018) and SOD2 (Candas et al., 2013; Jin et al., 2015), mouse SOD2 (Candas et al., 2013), or yeast SOD1 (Leitch et al., 2012; Tsang et al., 2014), the phosphorylation of SOD isoforms in plants has not been approved by genetic means so far. Mammalian SOD1 is phosphorylated at Thr2 (Fay et al., 2016) and Thr40 (Tsang et al., 2018), having a pronounced impact on its activity. For example, the mammalian target of rapamycin complex 1 (mTORC1) is a negative regulator of SOD1 activity under nutrient-rich conditions by reversible phosphorylation at Thr40 (Tsang et al., 2018). Yeast SOD1 is phosphorylated at Ser38 under low oxygen conditions, which allows its interaction with CCS and, consequently, proper folding and activation (Leitch et al., 2012). Contrarily, the double phosphorylation of yeast SOD1 at Ser60 and Ser99 by a DNA damage checkpoint kinase Dun1 during oxidative stress leads to the translocation of this SOD to the nucleus, where it binds to the promoters and activates ROS-responsive and DNA repair genes (Tsang et al., 2014). Recently, the first *in planta* evidence of the nuclear localization of SODs was reported in plants (Dvořák et al., 2020). Thus, in addition to well-known protective functions during oxidative stress, plant SODs might have similar functions in the nucleus as yeast SOD1 (Figure 5). However, this working hypothesis needs to be experimentally tested in the future. Rarely, phosphoproteomic studies have reported on the detection of phosphorylated amino acid residues in plant SODs. A gel-based study on mitochondrial phosphoproteomes identified phosphorylated MSD1 (Bykova et al., 2003). In addition, CSD2 and CSD3 were found to be phosphorylated in response to auxin and isoxaben, respectively (Zhang et al., 2013; Table 1). Multiple phosphorylation sites were also detected in FSD2 after ABA treatment (Wang et al., 2013b). These findings demonstrate that the phosphorylation of plant SODs may have some important functions that need to be elucidated in future studies.

On the other side, there is quite rich phosphoproteomic evidence on the phosphorylation of CAT isoforms in plants (Table 1). CAT phosphorylation has been broadly documented in human research (Kumar et al., 2010; Rafikov et al., 2014). A recent study suggested that high light-induced H_2_O_2_ is regulated by the interaction between CAT isoforms and BRASSINOSTEROID-INSENSITIVE 1 ASSOCIATED RECEPTOR KINASE 1 (BAK1), leading to CAT1 phosphorylation and activation (Zhang S. et al., 2020). CAT3 activity is enhanced through phosphorylation by CPK8, leading to H_2_O_2_ decomposition under drought stress (Zou et al., 2015). In rice, the CATC isoform is phosphorylated by SALT TOLERANCE RECEPTOR-LIKE CYTOPLASMIC KINASE 1 (STRK1), which acts as a positive regulator of salt and oxidative stress tolerance (Zhou et al., 2018). STRK1 is anchored in the plasma membrane *via* palmitoylation, where it interacts and phosphorylates the CATC isoform at Tyr210 (Zhou et al., 2018).

Notably, H_2_O_2_ decomposition is controlled by phosphorylation also within the ascorbate–glutathione cycle. Based on phosphoproteomic data, the stromal and thylakoid APX and MDAR1, MDAR2, and GR2 are enzymes prone to phosphorylation (Table 1). Thylakoid APX is negatively regulated by WHEAT KINASE START1 (WKS1)-dependent phosphorylation in wheat infected by stripe rust-inducing fungi *Puccinia striiformis* f. sp. *tritici*. Such decreased activity of thylakoid APX leads to the enhanced accumulation of peroxides, causing cell death upon pathogen attacks (Gou et al., 2015).

## Conclusion and Future Prospects

**Figure 6:**
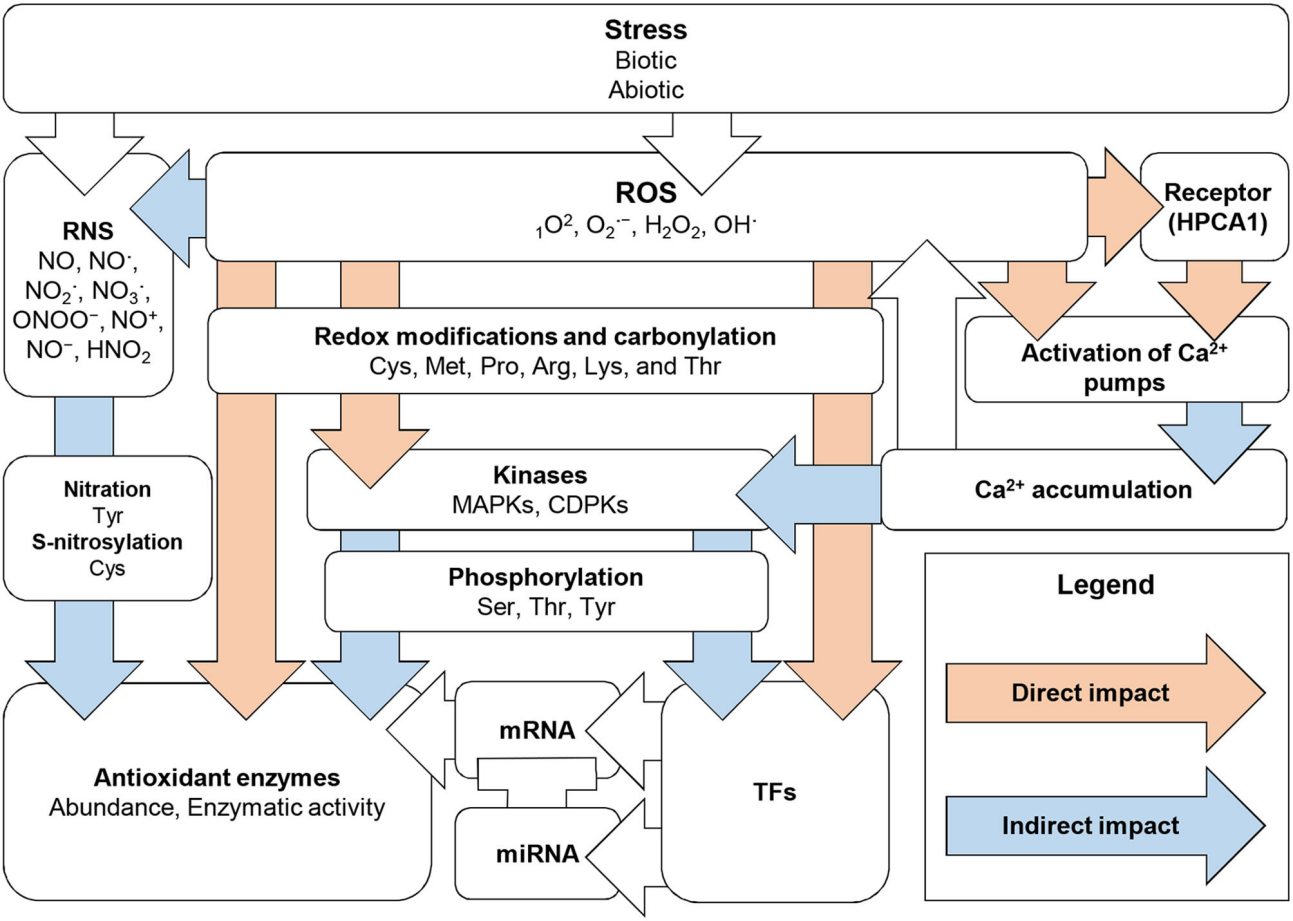
Overview of direct and indirect mechanisms of antioxidant enzyme regulation in response to stress-induced ROS generation.

In summary, the expression and activities of antioxidant enzymes are controlled both directly and indirectly at multiple levels with the involvement of ubiquitous secondary messengers (ROS, RNS, and Ca^2+^), PTMs (phosphorylation and redox-dependent ones), TFs, and other precise mechanisms (Figure 6). Currently, data on the coordination of the transcriptional and post-translational regulation of these enzymes are scarce. Moreover, the impact of protein–protein interactions on the functionality of antioxidant enzymes should not be underestimated. The complexity is increased by the requirement for rapid and spatially-specific antioxidant activation, which has to occur in subcellular- and tissue-dependent manners.

Further proteomic, genetic, cell, and molecular biology integrative investigations are necessary to uncover precise spatiotemporal regulations of individual antioxidant enzymes during plant development and stress responses. In addition, up-to-date proteomic, phospho- and redox-proteomic approaches might uncover new MAPK and CDPK targets modulating antioxidant defense during oxidative stress. The use of these techniques on transgenic plant lines with modified abundances of certain antioxidant enzymes may significantly contribute to the elucidation of their developmental and stress-related functions.

## Author Contributions

PD and AZ performed the bioinformatic prediction. PD and YK drafted the manuscript, which was revised and edited by TT and JŠ. All authors approved the final version of the manuscript.

## Conflict of Interest

The authors declare that the research was conducted in the absence of any commercial or financial relationships that could be construed as a potential conflict of interest.
